# Natriuresis During an Acute Intravenous Sodium Chloride Infusion in Conscious Sprague Dawley Rats Is Mediated by a Blood Pressure-Independent α_1_-Adrenoceptor-Mediated Mechanism

**DOI:** 10.3389/fphys.2021.784957

**Published:** 2022-01-17

**Authors:** Alissa A. Frame, Kayla M. Nist, Kiyoung Kim, Jill T. Kuwabara, Richard D. Wainford

**Affiliations:** ^1^Department of Pharmacology & Experimental Therapeutics, Boston University School of Medicine, Boston, MA, United States; ^2^Whitaker Cardiovascular Institute, Boston University School of Medicine, Boston, MA, United States; ^3^Department of Anatomy & Neurobiology, Boston University School of Medicine, Boston, MA, United States

**Keywords:** natriuresis, renal sympathetic nerves, adrenoceptors, sodium homeostasis, pressure-natriuresis

## Abstract

The mechanisms that sense alterations in total body sodium content to facilitate sodium homeostasis in response to an acute sodium challenge that does not increase blood pressure have not been fully elucidated. We hypothesized that the renal sympathetic nerves are critical to mediate natriuresis via α_1_- or β-adrenoceptors signal transduction pathways to maintain sodium balance in the face of acute increases in total body sodium content that do not activate the pressure-natriuresis mechanism. To address this hypothesis, we used acute bilateral renal denervation (RDNX), an anteroventral third ventricle (AV3V) lesion and α_1_- or β-antagonism during an acute 1M NaCl sodium challenge in conscious male Sprague Dawley rats. An acute 1M NaCl infusion did not alter blood pressure and evoked profound natriuresis and sympathoinhibition. Acute bilateral RDNX attenuated the natriuretic and sympathoinhibitory responses evoked by a 1M NaCl infusion [peak natriuresis (μeq/min) sham 14.5 ± 1.3 vs. acute RDNX: 9.2 ± 1.4, *p* < 0.05; plasma NE (nmol/L) sham control: 44 ± 4 vs. sham 1M NaCl infusion 11 ± 2, *p* < 0.05; acute RDNX control: 42 ± 6 vs. acute RDNX 1M NaCl infusion 25 ± 3, *p* < 0.05]. In contrast, an AV3V lesion did not impact the cardiovascular, renal excretory or sympathoinhibitory responses to an acute 1M NaCl infusion. Acute i.v. α_1_-adrenoceptor antagonism with terazosin evoked a significant drop in baseline blood pressure and significantly attenuated the natriuretic response to a 1M NaCl load [peak natriuresis (μeq/min) saline 17.2 ± 1.4 vs. i.v. terazosin 7.8 ± 2.5, *p* < 0.05]. In contrast, acute β-adrenoceptor antagonism with i.v. propranolol infusion did not impact the cardiovascular or renal excretory responses to an acute 1M NaCl infusion. Critically, the natriuretic response to an acute 1M NaCl infusion was significantly blunted in rats receiving a s.c. infusion of the α_1_-adrenoceptor antagonist terazosin at a dose that did not lower baseline blood pressure [peak natriuresis (μeq/min) sc saline: 18 ± 1 vs. sc terazosin 7 ± 2, *p* < 0.05]. Additionally, a s.c. infusion of the α_1_-adrenoceptor antagonist terazosin further attenuated the natriuretic response to a 1M NaCl infusion in acutely RDNX animals. Collectively these data indicate a specific role of a blood pressure-independent renal sympathetic nerve-dependent α_1_-adrenoceptor-mediated pathway in the natriuretic and sympathoinhibitory responses evoked by acute increases in total body sodium.

## Introduction

Maintenance of fluid and electrolyte homeostasis, which is essential for life, is dependent on neural, humoral, and hemodynamic mechanisms that alter renal sodium excretion in response to changes in total body sodium. Based on the classical model of “pressure-natriuresis,” it has been hypothesized that increases in total body sodium always evoke elevations in fluid volume and arterial blood pressure that raise renal perfusion pressure, ultimately initiating natriuresis and a return to sodium homeostasis (Guyton, 1991). However, recent studies performed in human subjects and animal models, suggest that increased renal sodium excretion, natriuresis, can occur in the absence of detectable changes in arterial blood pressure in response to mild salt loading (Kompanowska-Jezierska et al., 2008; Bie, 2009; Wainford et al., 2013). Although the integrated mechanisms that facilitate sodium homeostasis independently of activation of the pressure-natriuresis mechanism are poorly understood it has been suggested that suppression of the renin-angiotensin system and renal sympathetic nerve activity are critical to facilitate the natriuretic responses activated to maintain sodium balance (DiBona and Kopp, 1997; Manunta et al., 2011; Wainford et al., 2013). The renal sympathetic nerves directly regulate renal sodium handling through several mechanisms, including activation of renal β_1_-adrenergic receptors, resulting in renin release, and stimulation of renal α_1_-adrenergic receptors, resulting in sodium reabsorption (Persson et al., 1989; DiBona and Kopp, 1997). However, the mechanisms by which the renal sympathetic nerves influence the acute natriuretic response to increases in total body sodium in the absence of detectable changes in arterial blood pressure have not been fully elucidated.

Our laboratory has delineated a sympathoinhibitory renal sympathetic nerve-dependent pathway that facilitates sodium homeostasis independently of the renin-angiotensin-aldosterone system in response to an acute isovolumetric NaCl challenge that does not increase arterial blood pressure in conscious male Sprague Dawley rats (Wainford et al., 2013). In our prior study using this approach, we did not assess the role of the anteroventral third ventricle (AV3V) region which contains osmo- and sodium-sensitive neurons and can modulate sympathetic outflow (Toney et al., 2003; Stocker et al., 2010, 2013b) or the direct role of α_1_- or β-adrenoceptors in the observed acute natriuretic response. In the present study, we hypothesized that a renal sympathetic nerve-dependent α_1_- or β-adrenoceptor-mediated pathway regulates the acute natriuretic response to increases in total body Nacl independently of the pressure-natriuresis mechanism. To address this hypothesis, we: (1) examined the role of the AV3V region and the renal sympathetic nerves in acute NaCl-evoked natriuresis that occurs independent of detectable increases in blood pressure and activation of the pressure-natriuresis mechanism and (2) administered an acute sodium challenge during pharmacological α_1_- and β-adrenoceptor antagonism.

## Materials and Methods

### Ethical Approval

All animal protocols were approved by the Institutional Animal Care and Use Committee (IACUC) in accordance with the guidelines of Boston University School of Medicine and the National Institutes of Health *Guide for the Care and Use of Laboratory Animals* under IACUC protocol number PROTO201800201. As detailed in our surgical procedures, all steps possible were taken to minimize pain and suffering. Additionally, all animal studies detailed in this manuscript fully comply with the ethical principles of Frontiers in Physiology.

### Animals

Male Sprague Dawley rats aged 3-month-old, weighing 275–299 g, were purchased from Envigo (Indianapolis, Indiana, United States) for use in these studies. Prior to surgical intervention animals were pair-housed and were then housed individually post-surgery. Animals were housed in the Laboratory Animal Science Center at Boston University under a 12 h:12 h light:dark cycle under temperature (20–26°C) and humidity (30–70%) controlled conditions. Animals were provided tap water and standard irradiated rodent diet *ad libitum* [Envigo Teklad, WI, Teklad Global Diet #2918: 18% protein, 5% crude fat, 5% fiber, 0.6% K^+^ content, with a total NaCl content of 0.6% (174 mEquiv Na^+^ kg)]. All rats were randomly assigned to experimental groups.

### Surgical Procedures

#### Acute Femoral Vein, Femoral Artery, and Bladder Cannulation

On the day of the acute study, rats were anesthetized with sodium methohexital (20 mg kg^–1^ I.P., supplemented with 10mg kg^–1^ I.V. as required during surgery) (Wainford et al., 2013; Carmichael et al., 2016; Walsh et al., 2016). Prior to a surgical incision the area was injected with bupivacaine (2 mg/kg s.c.). Following the induction of anesthesia, the depth of anesthesia was confirmed by lack of response to a toe pinch. During all surgical procedures the depth of anesthesia was monitored continually by assessment of absence of a toe pinch response, observation of respiration, observation of mucous membranes and observation of any reaction to a surgical manipulation. Rats were instrumented with catheters in the left femoral vein, left femoral artery, and bladder to allow I.V. administration of isotonic saline, acute sodium challenges, and adrenoceptor antagonists, measurement of mean arterial pressure (MAP) and heart rate (HR), and assessment of renal function, respectively (Kapusta et al., 2012; Wainford et al., 2013; Carmichael et al., 2016; Walsh et al., 2016). Rats were placed in a Plexiglass rat holder and an I.V. infusion of isotonic saline (20 μL min^–1^) was maintained during a 2-h surgical recovery period, allowing rats to return to full consciousness and stable renal and cardiovascular function prior to experimentation. Placement of the surgically instrumented rat in a Plexiglass holder that facilities the collection of stable cardiovascular parameters and urine and allows the rat forward and backward movement and does not restrain the rat. This experimental approach increases the rigor and reproducibility of our studies as it parallels that conducted in multiple studies conducted in our laboratory, including our prior study utilizing 1M NaCl infusion (Wainford et al., 2013). Mean arterial pressure and HR were recorded continuously via the surgically implanted femoral artery cannula using computer-driven BIOPAC data acquisition software (MP150 and AcqKnowledge 3.8.2, CA) connected to an external pressure transducer (P23XL; Viggo Spectramed Inc., CA) (Wainford et al., 2013; Carmichael et al., 2016; Walsh et al., 2016; Moreira et al., 2019). The arterial cannula was flushed with small volumes of 0.9% NaCl following collection of arterial blood samples as described in the experimental protocols. Following completion of acute experimental studies, as described below, all animals were euthanized by I.V. injection of sodium thiopental (20 mg/kg) in accordance with stated IAUCUC approval. Death was confirmed via (1) absence of heart rate as assessed by BIOPAC software, and (2) opening of the chest cavity.

#### Intracerebroventricular Cannulation

Seven to 10 days before the day of the acute study, a subset of rats, some of whom had previously undergone an AV3V lesion, were anesthetized with ketamine (30mg kg^–1^ I.P.) in combination with xylazine (3mg kg^–1^ IP). The depth of anesthesia was confirmed as described above. A stainless steel cannula was stereotaxically implanted into the right lateral cerebral ventricle (Wainford and Kapusta, 2009; Wainford et al., 2013; Carmichael et al., 2016; Walsh et al., 2016). Rats were placed in their home cages and monitored during surgical recovery.

#### AV3V Lesion

Twenty-five days before the day of acute study, a subset of rats were anesthetized with ketamine (30mg kg^–1^ I.P.) in combination with xylazine (3mg kg^–1^ I.P.). The depth of anesthesia was confirmed as described above. An anodal electrolytic lesion (2.5 mA for 25 s) was stereotaxically delivered to the AV3V [stereotaxic coordinates: 0.3 mm posterior to bregma, on midline, 7.5 mm ventral to the midsagittal sinus (Simmonds et al., 2014)] using an insulated 23-g nichrome wire exposed only at the tip. In a separate sham group, the nichrome wire was inserted 4 mm into the brain for 25s but no lesion was delivered. Rats were placed in their home cages and monitored during surgical recovery and received s.c. buprenorphine (0.03mg/kg) for 48-h post-surgery. AV3V lesion was verified by postlesion adipsia assessed as fluid intake less than 5 mL during the first 24 h post-lesion (Callahan et al., 1988) and the absence of a pressor response to i.c.v. Ang II (200 ng) (Moreira et al., 2009; Vieira et al., 2013) was assessed post-1M NaCl infusion study in a subset of rats instrumented with an i.c.v. cannula. Adipsic rats were given 5% sucrose water *ad libitum* to encourage drinking and gradually weaned to normal water over 5-days prior to assignment to an experimental study group.

#### Acute Bilateral Renal Denervation

In a subset of rats immediately following cannulation of the femoral vein, femoral artery, and bladder bilateral acute bilateral renal denervation (RDNX) was performed using standard techniques prior to the surgical recovery period. In brief, anesthesia was maintained with sodium methohexital (10mg kg^–1^ I.V. as required) and each kidney was exposed via a dorsal flank incision. The renal vein and artery were dissected from the surrounding fascia and stripped of visible nerve bundles. The renal artery and any visible nerve fibers were coated with a 10% phenol solution in ethanol to destroy any remaining nerve fibers. In a separate sham group, a similar surgical procedure was performed but nerves bundles were not disrupted or treated with phenol (Kapusta et al., 2013; Wainford et al., 2013, 2015; Carmichael et al., 2020). Rats were allowed to recover as described above prior to experimentation. The efficacy of acute RDNX was confirmed at the end of the study via ELISA analysis of norepinephrine (NE) content in kidney tissue as per the manufacturer’s instructions (IB89537, IBL America, Minneapolis, MN, United States).

#### Subcutaneous Osmotic Minipump Implantation

Rats were anesthetized using sodium brevital (20 mg/kg IP) and were randomly assigned to be surgically implanted with an osmotic minipump (2ML4, Alzet) subcutaneously in the subscapular region (Walsh et al., 2016; Frame et al., 2019b) delivering a continuous 21-day s.c. infusion (2.5 μL/h) of 50/50 isotonic saline/DMSO or terazosin hydrochloride [10mg/kg/day (Maranon et al., 2015; Frame et al., 2019b) Sigma] dissolved in 50/50 saline/DMSO (Frame et al., 2019b; Puleo et al., 2020).

#### Acute Experimental Protocols

##### I.V. 1M NaCl Infusion

Following a 2h surgical recovery period, groups of intact, sham RDNX, bilateral RDNX, sham AV3V lesion and AV3V lesion rats (*N* = 6/group) underwent an acute 1M NaCl infusion protocol. The 5-h protocol consists of a 1-h control period (isotonic saline, 20 μL min^–1^, I.V.) followed by a 2-h 1M NaCl infusion (1M NaCl, 20 μL min^–1^, I.V.) and a subsequent 2-h recovery period (isotonic saline, 20 μL min^–1^, I.V.) (Kompanowska-Jezierska et al., 2008; Wainford et al., 2013). Mean arterial pressure and HR were monitored continuously and urine was collected in consecutive 15-min increments throughout the protocol. Arterial blood samples were collected at the start of the final 15-min increment of the control, 1M NaCl infusion and recovery periods for the measurement of hematocrit, EBV, EPV, plasma norepinephrine and plasma Na^+^ (Wainford et al., 2013). To allow for the assessment of glomerular filtration rate (GFR) and renal blood flow (RBF) during the 1M NaCl infusion, a separate group of intact rats were infused with inulin (300 mg kg^–1^h^–1^) and para-aminohippurate (PAH; 40 mg kg^–1^h^–1^) for a 90-min equilibration period during the 2h surgical recovery period and continuing throughout the 5-h study protocol described above. Urine was collected in consecutive 15-min increments and blood was collected in the middle of each half hour period during the study protocol.

##### I.V. 1M NaCl Infusion During Acute α_1_- or β-Adrenoceptor Antagonism

Following surgical recovery, groups of rats underwent a 1-h control period (isotonic saline, 20μL min^–1^, I.V.) after which an I.V. infusion of an α_1_-adrenoceptor antagonist (terazosin, 4.17 μg kg^–1^ min^–1^; intact, ADNX and RDNX rats) or β-adrenoceptor antagonist (propranolol, 6.94 μg kg^–1^ min^–1^; intact rats only) (*N* = 6/group) was initiated at the same 20 μL min^–1^ rate and was maintained throughout the protocol for an additional 1-h blockade baseline period immediately prior to a 2-h 1M NaCl co-infusion (1M NaCl, 20 μL min^–1^, I.V.) and a 2-h recovery period. Mean arterial pressure and HR were recorded continuously and urine was collected in consecutive 15-min increments throughout the protocol. Arterial blood samples were collected at the start of the final 15-min increment of the control, blockade baseline, 1M NaCl infusion, and recovery periods for the measurement of hematocrit, EPV, EBV and plasma Na^+^. After completion of the protocol, phenylephrine (4 μg bolus, I.V.) and isoproterenol (0.7 μg bolus, I.V.) were administered to confirm selective adrenoceptor blockade (Veitenheimer et al., 2012).

##### I.V. 1M NaCl Infusion During Chronic s.c.α_1_-Adrenoceptor Antagonism

Following surgical recovery, groups of rats that have received a 21-day s.c. osmotic minipump infusion of vehicle or terazosin, and groups of rats that underwent acute RDNX on the morning of the acute study underwent an I.V. 1M NaCl infusion study as described above. After completion of the protocol, phenylephrine (4 μg bolus, I.V.) and isoproterenol (0.7 μg bolus, I.V.) were administered to confirm selective adrenoceptor blockade (Veitenheimer et al., 2012).

##### Analytical Techniques

Pulsatile Arterial Pressure (PAP) was assessed using the Hemodynamic Analysis parameter for peak systolic and diastolic pressure events using AcqKnowledge 3.8.2 software. This analysis was conducted on the continuous arterial blood pressure signal recorded in the 20-min control baseline period in all studies. Signals that exhibited a strong noise characteristic, as defined by the Hemodynamic Analysis Parameter were excluded from analysis. Urine volume was assessed gravimetrically assuming 1 g = 1 mL. Urine and plasma sodium content were determined by flame photometry (IL-943; Instrumentation Laboratory, Bedford, MA, United States). Hematocrit (Hct) was determined using a micro-hematocrit centrifuge (Adams Readacrit, Clay Adams, NJ). Estimated plasma volume (EPV) was calculated using the following equation: EPV = [0.065 × body weight (kg)] × (1 – Hct). Plasma NE content was determined via ELISA (Immuno-Biological Laboratories, Minneapolis, MN; IB89552) following the manufacturer’s instructions. Hct was used to calculate estimated plasma volume (EPV) and estimated blood volume (EBV) using the following equations; EPV = [0.065 × body weight (kg)] × (100 – Hct), EBV = (EPV × 100)/(100-Hct).

### Statistical Analysis

All data are expressed as mean ± SEM. The normal (Gaussian) distribution of the data was assessed by a Kolmogorov-Smirnow test. The magnitude of change in cardiovascular and renal excretory parameters at different time points after initiation of a 1M NaCl sodium infusion was compared with the average group control value by a one-way repeated-measures analysis of variance (ANOVA) with subsequent Dunnett’s test. Differences between treatment groups (e.g., sham RDNX vs. acute RDNX) were assessed by a two-way repeated measure ANOVA with treatment group being one fixed effect and time the other, with the interaction included. The time (minutes) was used as the repeated factor. *Post hoc* analysis was performed using Bonferroni’s test. In all studies, statistical significance was defined as *P* < 0.05. All statistical analyses were performed using Graphpad (GraphPad Prism v.8 for Mac OS X, GraphPad Software, San Diego, CA, United States).

## Results

### Effect of an AV3V Lesion on the Cardiovascular, Renal, and Sympathoinhibitory Responses to a Non-pressor 1M NaCl Infusion

**FIGURE 1:**
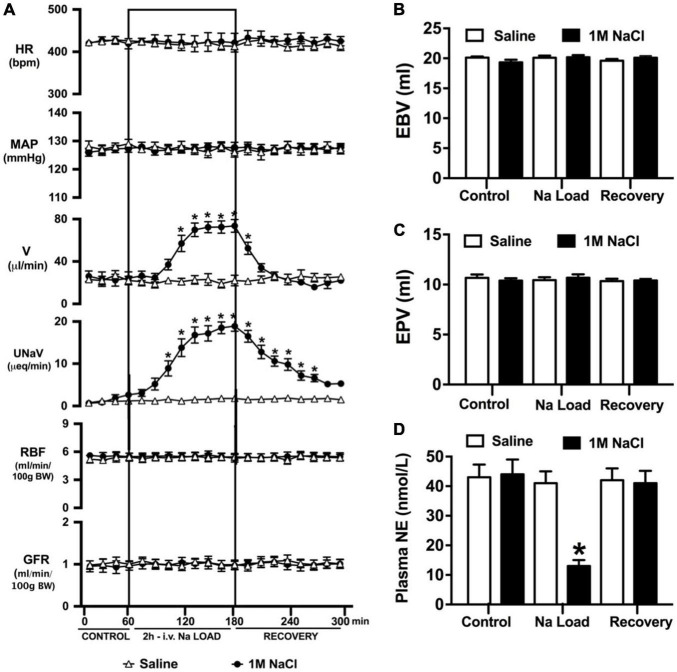
Cardiovascular and renal responses to an acute 1M NaCl infusion **(A)** Cardiovascular and renal responses, **(B)** Estimated blood volume (ml), **(C)** Estimated plasma volume (ml), and **(D)** Plasma norepinephrine content (nmol/L) during a 1-h control isotonic saline infusion (20 μl/min), a 2-h 1M NaCl infusion (20 μl/min) and a 2-h recovery period of isotonic saline infusion (20 μl/min) in conscious male Sprague Dawley rats. HR = heart rate (bpm), MAP = mean arterial pressure (mmHg), V = urinary flow rate (μL/min), UNaV = urinary sodium excretion (μeq/min), GFR = glomerular filtration rate [ml/min/100g body weight (BW)], RPF = estimated renal plasma flow (ml/min/100 g BW). Data are presented as mean ± SEM, *N* = 6/group. *p < 0.05 vs. group baseline value in last 15-min of the control saline infusion.

**FIGURE 2:**
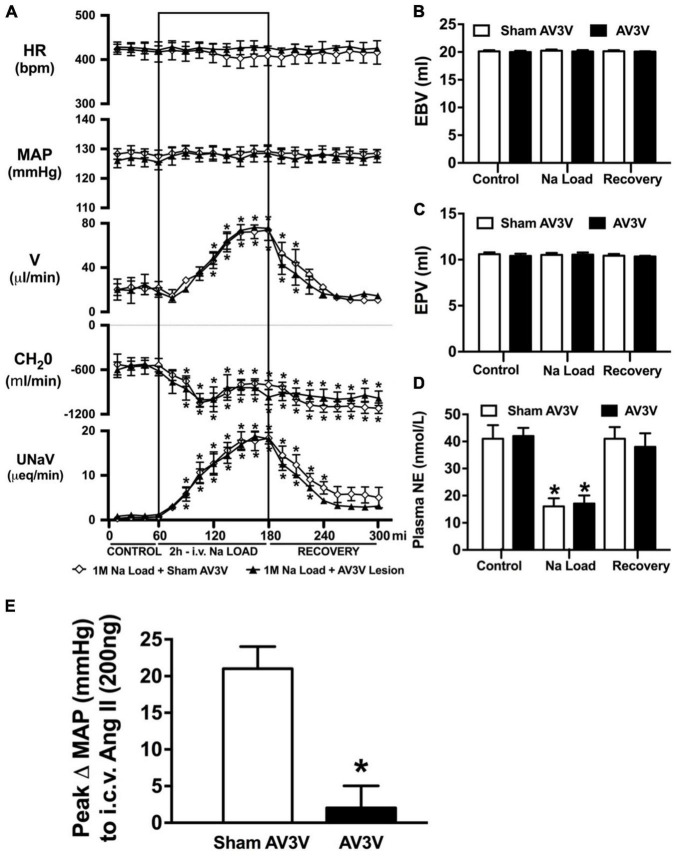
Effect of an AV3V lesion on the cardiovascular and renal responses to an acute 1M NaCl infusion **(A)** Cardiovascular and renal responses, **(B)** Estimated blood volume (ml), **(C)** Estimated plasma volume (ml), and **(D)** Plasma norepinephrine content (nmol/L) during a 1-h control isotonic saline infusion (20 μl/min), a 2-h 1M NaCl infusion (20 μl/min) and a 2-h recovery period of isotonic saline infusion (20 μl/min) in sham or AV3V lesioned conscious male Sprague Dawley rats, and **(E)** peak Δ MAP following an i.c.v. bolus injection of Ang II (200 ng) *N* = 4/group for Ang II administration. HR = heart rate (bpm), MAP = mean arterial pressure (mmHg), V = urinary flow rate (μL/min), CH_2_O = free water clearance, UNaV = urinary sodium excretion (μeq/min). Data are presented as mean ± SEM, *N* = 8/group except where indicated. *p < 0.05 vs. group baseline value in last 15-min of the control saline infusion.

An acute 2-h 1M NaCl infusion did not alter MAP or HR at any time point in naïve rats (Figure 1). In naïve rats, and all other treatment groups, we observed a PAP of approximately 30 mmHg (Table 1). In terms of renal function, a 1M NaCl infusion evoked a profound natriuretic and diuretic response without altering GFR or RBF (Figure 1). Further, in these same animals a 1M NaCl infusion evoked suppression of plasma NE (Figure 1D) but did not alter plasma sodium levels [Plasma Na^+^ (mEq/L); 0.9% Saline Infusion: Control 139.6 ± 0.4, 0.9% saline infusion 140.1 ± 0.5, Recovery 139.9 ± 0.5; 1M NaCl Infusion: Control 139.2 ± 0.6, 1M NaCl infusion140.4 ± 0.4, Recovery 139.8 ± 0.4], EBV or EPV (Figures 1B,C). An AV3V lesion had no impact on baseline cardiovascular and renal parameters or the physiological effects elicited by a 1M NaCl infusion compared to those observed in sham AV3V lesioned rats (Figure 2). In both groups, sham and AV3V lesioned, a 1M NaCl infusion had no impact on MAP, HR, plasma sodium, EPV or EBV, and resulted in significant diuresis, natriuresis and sympathoinhibition (Figure 2). AV3V lesions were confirmed by the observation of post-lesion adipsia in all animals [24h Fluid Intake post-lesion (ml); AV3V lesion 3.6 ± 0.7ml vs. Sham lesion 13.1 ± 0.6ml, *P* < 0.05, N = 8/group]. A subset of animals received an acute i.c.v. bolus injection of Ang II (200ng) following completion of the 1M NaCl infusion (*N* = 4/group). In sham animals we observed a significant pressor response to Ang II which was absent in all AV3V lesioned rats (Figure 2E).

**TABLE 1:** Mean arterial pressure (MAP; mmHg), Systolic Blood Pressure (SBP; mmHg), Diastolic Blood Pressure (DBP, mmHg) and Pulsatile Arterial Pressure calculated as SBP – DBP (PAP; mmHg) assessed during the 20-min control baseline period in naïve, sham anteroventral third ventricle (AV3V) lesion, AV3V lesion, sham renal denervated (RDNX), acutely RDNX, i.v. terazosin, i.v. propranolol, s.c. saline, s.c. terazosin and s.c. terazosin + acutely RDNX male Sprague Dawley rats for which physiological data in presented in Figures 1–7.

	MAP (mmHg)	SBP (mmHg)	DBP (mmHg)	PAP (mmHg)
Naïve	127 ± 2	139 ± 4	109 ± 4	28 ± 3
Sham AV3V	128 ± 2	144 ± 4	111 ± 3	32 ± 3
AV3V lesion	126 ± 3	142 ± 4	116 ± 4	28 ± 3
Sham RDNX	129 ± 2	141 ± 3	110 ± 3	32 ± 2
RDNX	128 ± 2	141 ± 5	112 ± 4	29 ± 3
i.v. terazosin	126 ± 4	139 ± 6	112 ± 7	26 ± 4
i.v, propranolol	127 ± 6	139 ± 5	106 ± 7	28 ± 5
s.c. saline	124 ± 3	137 ± 5	107 ± 4	31 ± 4
s.c. terazosin	123 ± 4	134 ± 4	105 ± 3	28 ± 5
s.c terazosin + RDNX	124 ± 2	140 ± 3	113 ± 4	27 ± 3

*Data are presented as mean ± SEM, N = 6/group with the exception of N = 8/group in sham AV3V and AV3V lesion groups.*

**FIGURE 3:**
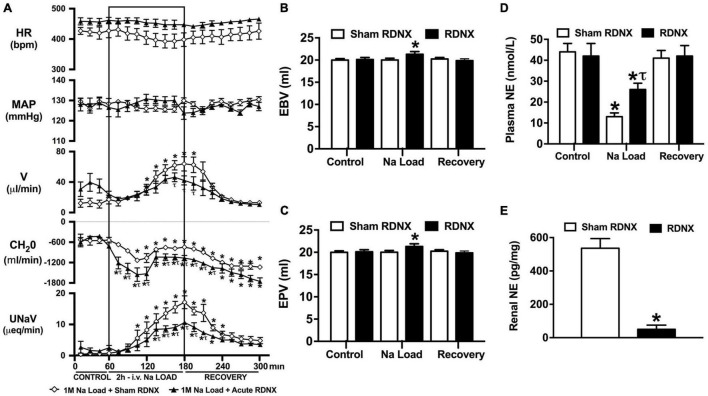
Effect of acute renal denervation (RDNX) on the cardiovascular and renal responses to an acute 1M NaCl infusion **(A)** Cardiovascular and renal responses, **(B)** Estimated blood volume (ml), **(C)** Estimated plasma volume (ml), and **(D)** Plasma norepinephrine content (nmol/L) during a 1-h control isotonic saline infusion (20 μl/min), a 2-h 1M NaCl infusion (20 μl/min) and a 2-h recovery period of isotonic saline infusion (20 μl/min) in sham or acutely RDNX conscious male Sprague Dawley rats, **(E)** renal NE content (pg/mg). HR = heart rate (bpm), MAP = mean arterial pressure (mmHg), V = urinary flow rate (μL/min), CH_2_O = free water clearance, UNaV = urinary sodium excretion (μeq/min). Data are presented as mean ± SEM, *N* = 6/group. *p < 0.05 vs. group baseline value in last 15-min of the control saline infusion. *τp* < 0.05 vs. respective sham RDNX group value.

**FIGURE 4:**
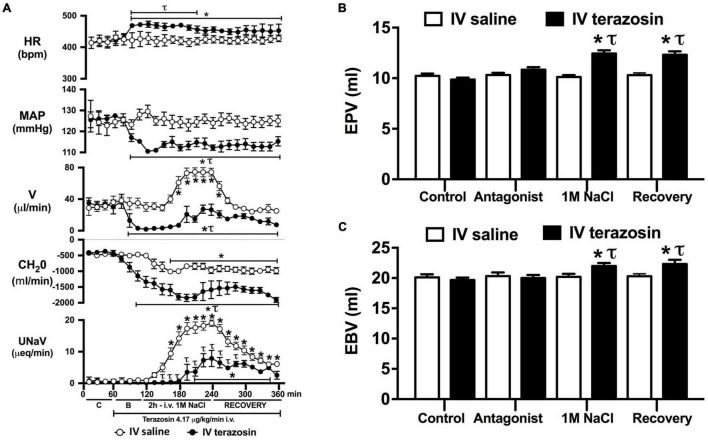
Effect of acute i.v. terazosin-mediated α_1_-drenoceptor antagonism on the cardiovascular and renal responses to an acute 1M NaCl infusion **(A)** Cardiovascular and renal responses, **(B)** Estimated blood volume (ml), and **(C)** Estimated plasma volume (ml) during a 1-h control isotonic saline infusion (20 μl/min), a 1-h baseline terazosin infusion (4.17 μg kg^–1^ min^–1^ in isotonic saline; 20 μl/min), a 2-h terazosin and 1M NaCl infusion (20 μl/min) and a 2-h recovery period of terazosin in isotonic saline infusion (20 μl/min) in intact conscious male Sprague Dawley rats, HR = heart rate (bpm), MAP = mean arterial pressure (mmHg), V = urinary flow rate (μL/min), CH_2_O = free water clearance, UNaV = urinary sodium excretion (μeq/min), C = control saline infusion, B = Baseline during terazosin infusion. Data are presented as mean ± SEM, *N* = 6/group. *p < 0.05 vs. group baseline value in last 15-min of the control saline infusion. *τp* < 0.05 vs. respective isotonic saline group infusion value.

**FIGURE 5:**
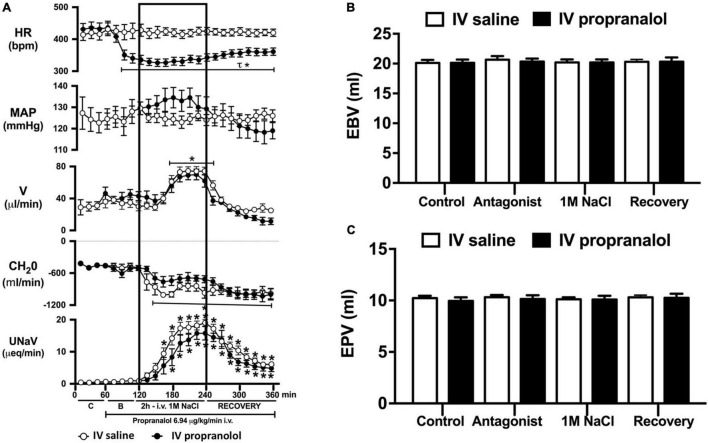
Effect of acute i.v. propranolol-mediated β-adrenoceptor antagonism on the cardiovascular and renal responses to an acute 1M NaCl infusion **(A)** Cardiovascular and renal responses, **(B)** Estimated blood volume (ml), and **(C)** Estimated plasma volume (ml) during a 1-h control isotonic saline infusion (20 μl/min), a 1-h baseline propranolol infusion (6.94 μg kg^–1^ min^–1^ in isotonic saline; 20 μl/min), a 2-h propranolol and 1M NaCl infusion (20 μl/min) and a 2-h recovery period of propranolol in isotonic saline infusion (20 μl/min) in intact conscious male Sprague Dawley rats. HR = heart rate (bpm), MAP = mean arterial pressure (mmHg), V = urinary flow rate (μL/min), CH_2_O = free water clearance, UNaV = urinary sodium excretion (μeq/min). Data are presented as mean ± SEM, *N* = 6/group. *p < 0.05 vs. group baseline value in last 15-min of the control saline infusion. *τp* < 0.05 vs. respective isotonic saline group infusion value. Please note the same isotonic saline group data is presented in Figures 4, 5 for clarity.

**FIGURE 6:**
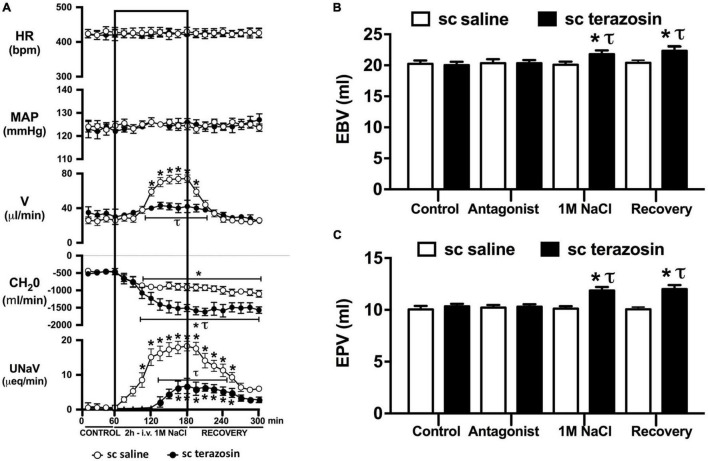
Effect of chronic s.c. terazosin-mediated α_1_-adrenoceptor antagonism on the cardiovascular and renal responses to an acute 1M NaCl infusion **(A)** Cardiovascular and renal responses, **(B)** Estimated blood volume (ml), and **(C)** Estimated plasma volume (ml) during a 1-h control isotonic saline infusion (20 μl/min), a 2-h 1M NaCl infusion (20 μl/min) and a 2-h recovery period of isotonic saline infusion (20 μl/min) in intact conscious male Sprague Dawley rats receiving a s.c. saline/DMSO vehicle or s.c. terazosin (10 mg/kg/day) infusion. HR = heart rate (bpm), MAP = mean arterial pressure (mmHg), V = urinary flow rate (μL/min), CH_2_O = free water clearance, UNaV = urinary sodium excretion (μeq/min). Data are presented as mean ± SEM, *N* = 6/group. *p < 0.05 vs. group baseline value in last 15-min of the control saline infusion. *τp* < 0.05 vs. respective sc saline/DMOS group infusion value.

**FIGURE 7:**
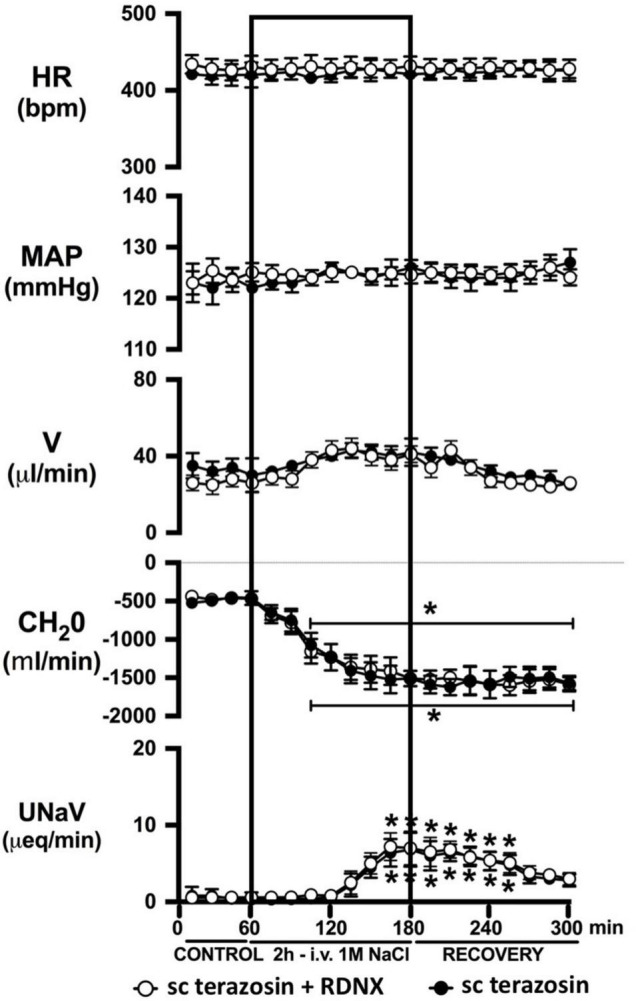
Effect of acute RDNX in combination with chronic s.c. terazosin-mediated 1-adrenoceptor antagonism on the cardiovascular and renal responses to an acute 1M NaCl infusion Cardiovascular and renal responses during a 1-h control isotonic saline infusion (20 μl/min), a 2-h 1M NaCl infusion (20 μl/min) and a 2-h recovery period of isotonic saline infusion (20 μl/min) in intact or acutely RDNX conscious male Sprague Dawley rats receiving a sc terazosin (10 mg/kg/day) infusion. HR = heart rate (bpm), MAP = mean arterial pressure (mmHg), V = urinary flow rate (μL/min), CH_2_O = free water clearance, UNaV = urinary sodium excretion (μeq/min). Data are presented as mean ± SEM, *N* = 6/group. *p < 0.05 vs. group baseline value in last 15-min of the control saline infusion. Please note the sc terazosin data in intact animals is transposed from Figure 6 for clarity.

### Effect of Acute Renal Denervation on the Cardiovascular, Renal, and Sympathoinhibitory Responses to a Non-pressor 1M NaCl Infusion

An acute 2-h 1M NaCl infusion did not alter MAP or HR at any time point in sham RDNX rats (Figure 3A). In sham RDNX rats, in which the renal sympathetic nerves are intact, a 1M NaCl infusion evoked profound natriuresis and diuresis, reduced free water clearance and evoked suppression of plasma NE content (Figures 3A,D). Further, in these same sham RDNX animals a 1M NaCl infusion did not alter plasma sodium levels [Plasma Na^+^ (mEq/L); 1M NaCl Infusion in Sham RDNX; Control 139.2 ± 0.6, 1M NaCl infusion 140.4 ± 0.4, Recovery 139.8 ± 0.4] or EBV and EPV (Figures 3B,C). In animals that underwent acute bilateral RDNX, similarly to naïve (Figure 1) and sham RDNX animals (Figure 3), a 1M NaCl infusion did not alter cardiovascular parameters. Acute bilateral RDNX attenuated the natriuretic and diuretic responses to a 1M NaCl infusion [peak UNaV (μeq min^–1^) sham 14.5 ± 1.3 versus. RDNX: 9.2 ± 1.4, *p* < 0.05; peak V (μL min^–1^) sham: 62.6 ± 10.1 versus. RDNX: 38.8 ± 8.8, *p* < 0.05] and attenuated 1M NaCl-evoked suppression of plasma NE [plasma NE (nmol/L) Sham RDNX: control: 44 ± 4 versus. 1M NaCl infusion 11 ± 2, *p* < 0.05; RDNX: control: 42 ± 6 versus. 1M NaCl infusion 25 ± 3] (Figure 3D). Additionally, acute bilateral RDNX resulted in short term increases in EBV and EPV during the 1M NaCl infusion that returned to baseline during the recovery period (Figures 3B,C). However, acute RDNX did not impact plasma sodium levels [Plasma Na^+^ (mEq/L); Control; Sham RDNX 139.6 ± 0.4 versus RDNX 140.1 ± 0.5, 1M NaCl infusion Sham RDNX 140.6 ± 0.5 versus RDNX 140.8 ± 0.5, Recovery Sham RDNX 139.9 ± 0.5 versus RDNX 140.3 ± 0.5]. The efficacy of acute bilateral RDNX was confirmed by assessment of renal norepinephrine content, in which renal NE content was reduced by approximately 90% in acutely denervated rats vs. sham RDNX animals (Figure 3E).

### Effect of Acute α_1_ vs. β-Adrenoceptor Antagonism on the Cardiovascular and Renal Responses to an Acute Non-pressor 1M NaCl Infusion

Acute α_1_-adrenoceptor antagonism reduced baseline MAP by approximately 20 mmHg and increased HR by approximately 40 bpm (Figure 4A). In terms of renal parameters there was a suppression of diuresis, free water clearance, with non-statistically significant reduction in natriuresis during acute α_1_-adrenoceptor antagonism. In contrast, acute β-adrenoceptor antagonism had no effect on MAP or renal excretory parameters and reduced baseline HR approximately 100 bpm (Figure 5A). During α_1_-adrenoceptor antagonism we observed no change in cardiovascular parameters but profound attenuation of the natriuretic and diuretic responses to a 1M NaCl infusion (Figure 4A). Further, α_1_-adrenoceptor antagonism resulted in an increase in EBV and EPV during the 1M NaCl infusion and recovery periods (Figures 4B,C) – an increase not observed in naïve 1M NaCl treated animals. As observed following acute RDNX we did not see any alteration in plasma sodium content during 1M NaCl infusion [Plasma Na^+^ (mEq/L); Control 140.2 ± 0.5, 1M NaCl infusion140.8 ± 0.6, Recovery 140.7 ± 0.6]. In contrast to profound renal effects observed of α_1_ blockade during a 1M NaCl infusion acute β-adrenoceptor antagonism had no effect on the natriuretic and diuretic responses to 1M NaCl infusion, and did not alter EPV (Figure 5). Confirming the selectivity and efficacy adrenoceptor antagonism (1) the average MAP response to an IV bolus of the α_1_-agonist phenylephrine was 22 ± 3 mmHg in propranolol treated rats vs. 2 ± 3 mmHg in terazosin treated rats, and (2) the average HR response to the β1-agonsit isoproterenol was 16 ± 7 bpm in propranolol treated rats vs. 95 ± 14 bpm in terazosin treated rats.

### Effect of Chronic α_1_-Adrenoceptor Antagonism on the Cardiovascular and Renal Responses to an Acute Non-pressor 1M NaCl Infusion

Chronic α_1_-adrenoceptor antagonism, via s.c. osmotic minipump infusion, replicating our prior data in young normotensive Sprague Dawley rats, had no impact on baseline cardiovascular or renal parameters (Figure 6A). As observed during acute i.v. antagonism of α_1_-adrenoceptors (Figure 5) during s.c. α_1_-adrenoceptor antagonism we observed no change in cardiovascular parameters during a 1M NaCl infusion. Further, s.c. α_1_-adrenoceptor antagonism resulted in significant attenuation of the natriuretic and diuretic responses to a 1M NaCl infusion (Figure 6A) and increased both EBV and EPV during the 1M NaCl infusion and recovery periods (Figures 6B,C). A 1M NaCl infusion did not alter plasma sodium in animals receiving a s.c. terazosin infusion [Plasma Na^+^ (mEq/L); Control 140.1 ± 0.3, 1M NaCl infusion 140.3 ± 0.4, Recovery 140.3 ± 0.5]. Acute RDNX (confirmed by reduced renal NE content) immediately prior to the 1M NaCl load had no significant effect impact on the physiological responses observed during α_1_-adrenoceptor antagonism (Figure 7). Confirming the selectivity and efficacy adrenoceptor antagonism in intact and RDNX rats (1) the average MAP response to an IV bolus of the α_1_-agonist phenylephrine was 24 ± 4 mmHg in s.c. vehicle infused rats (*N* = 6) vs. 1 ± 2 mmHg in s.c. terazosin infused rats (*N* = 12), and (2) the average HR response to the β1-agonsit isoproterenol was 98 ± 10 bpm in s.c. vehicle infused rats (*N* = 6) rats vs. 93 ± 12 bpm in s.c. terazosin infused rats.

## Discussion

The current studies were designed to delineate the role(s) of the renal sympathetic nerves and circumventricular organs, and α_1_- and β-adrenoceptors in the natriuretic responses evoked by an acute change in total body sodium content. Extending our prior studies, we demonstrate a central role of the renal sympathetic nerves, but not circumventricular organs, in mediating the natriuretic response to an acute 1M NaCl infusion that does not increase blood pressure in conscious Sprague Dawley rats (Wainford et al., 2013). Further, in this experimental paradigm in which 1M NaCl does not increase blood pressure, we demonstrate via acute antagonism that α 1-, but not β-adrenoceptors, are essential to mediate the natriuretic response and to maintain fluid balance. To address the potential confounding effect of acute α_1_-adrenoceptor antagonism evoking a drop in blood pressure we validated the role of α_1_-adrenoceptors on the natriuretic response to a 1M NaCl infusion in animals in which α_1_-adrenoceptors were chronically antagonized independent of a reduction in blood pressure. These data demonstrate a pivotal role of the renal sympathetic nerves and α_1_-adrenoceptors in the acute natriuretic responses triggered by increased total body sodium.

It is well established that multiple sites within the body detect alterations in plasma sodium and osmolality via osmo/sodium receptors to trigger natriuretic responses – including the sensory afferent renal sympathetic nerves and the circumventricular organs (Stocker et al., 2013a; Johns, 2014; Kopp, 2015). To investigate the potential role(s) of the renal sympathetic nerves and the circumventricular organs in the acute natriuretic response to increased total body sodium content we utilized a 1M NaCl infusion (Wainford et al., 2013). A 2-h 1M NaCl infusion in conscious male Sprague Dawley rats evoked profound natriuresis, diuresis and sympathoinhibition without altering mean arterial blood pressure, plasma volume, renal blood flow, glomerular filtration rate or plasma sodium levels. These data are consistent with the effects of this acute sodium challenge paradigm in conscious and anesthetized rats as previously reported by several laboratories, including our own (Roson et al., 2006, 2010; Kompanowska-Jezierska et al., 2008; Wainford et al., 2013). Based on the absence of detectable alterations in renal hemodynamics or arterial blood pressure, we conclude that the observed sympathoinhibitory and natriuretic responses evoked by a 1M NaCl infusion occur independent of activation of the pressure-natriuresis mechanism. As such, this experimental paradigm enables the study of the control of renal excretion in response to a 1M NaCl infusion in a conscious rat independent of changes in arterial blood pressure – as occurs physiologically except in the setting of excessive intake of salt. We acknowledge that a limitation of this experimental paradigm, utilized in all studies in this manuscript, is that basal blood pressure is elevated due to surgical stress and placement in a plexiglass holder to permit the simultaneous collection of cardiovascular and renal excretory parameters. There is the potential that this mildly elevated basal blood pressure may have prevented the detection of a 1M NaCl infusion evoked alteration in blood pressure. However, it should be noted that is experimental paradigm has been widely used by our laboratory and in several prior studies, in animals with similar baseline blood pressures, we have observed alterations in blood pressure in response to physiological and pharmacological stimuli, including an IV 3M NaCl bolus administration (Carmichael et al., 2016) – which strongly suggests that our observation of no change in blood pressure during a 1M NaCl infusion is valid and not dependent on the mild elevation in baseline blood pressure. Further, our observation of a PAP of approximately 30 mmHg in all experimental groups is in accordance with the PAP reported in normotensive rat strains and is not indicative of hypertensive level of PAP such as is observed hypertensive rats, e.g., the spontaneously hypertensive rat (Safar and Laurent, 2003; Letson et al., 2019).

In our prior study utilizing this experimental paradigm we reported that chronic bilateral RDNX, in which the renal sympathetic nerves were removed 10–14 days prior to study, had no impact on the natriuretic or sympathoinhibitory responses evoked by a 1M NaCl infusion (Wainford et al., 2013). However, multiple renal sympathetic nerve-independent mechanisms become activated to facilitate the restoration of fluid and electrolyte balance following RDNX (DiBona and Sawin, 1983), potentially masking a direct role of the renal sympathetic nerves in our prior study. To directly assess the impact of the renal sympathetic nerves on the physiological responses to a 1M NaCl load in the absence of compensatory mechanisms, we conducted acute bilateral RDNX immediately prior to the acute experimental protocol. Given the short time frame between removal of the renal nerves and the administration of the 1M NaCl sodium infusion, approximately 3 h, we believe this approach avoids the confounding effects of non-renal nerve-mediated compensatory homeostatic mechanisms that take several days to restore sodium and water balance (DiBona and Sawin, 1983). The efficacy of acute RNDX was confirmed by a significant reduction in renal norepinephrine content.

In accordance with our prior observations in Sprague Dawley rats that had intact renal sympathetic nerves or underwent chronic RDNX (Wainford et al., 2013), acute RDNX had no impact on baseline cardiovascular and renal parameters or baseline levels of circulating plasma NE. Further, we observed that following acute RDNX a 1M NaCl infusion did not alter blood pressure, heart rate or plasma sodium content. In these animals acute RDNX moderately blunted the diuresis, profoundly attenuated the natriuresis (peak natriuresis reduced approximately 50%) and attenuated the sympathoinhibitory response elicited by a 1M NaCl infusion. The absence of changes in heart rate or blood pressure during 1M NaCl infusion in intact or RDNX rats suggest that the observed alterations in circulating plasma NE do not reflect functional changes in sympathetic outflow to the heart or vasculature. Our observation of an attenuated natriuretic response, accompanied by increased estimated blood and plasma volume during an 1M NaCl infusion in acute RDNX rats, supports our hypothesis that the renal sympathetic nerves mediate a sympathoinhibitory pathway that modulates renal sodium excretion independently of the pressure-natriuresis mechanism. Based on our prior finding that selective ablation of the afferent renal nerves does not impact the physiological responses to a 1M NaCl infusion (Frame et al., 2019a) we speculate the observed sympathoinhibitory pathway is not mediated by the sympathoinhibitory afferent renal nerve mediated reno-renal reflex. Further, our finding that verified AV3V lesions, in which the osmo/sodium sensitive neurons of the circumventricular organs are ablated, have no impact on the natriuretic or sympathoinhibitory responses to a 2-h 1M NaCl infusion suggests afferent projections from osmosenstive forebrain structures (e.g., subfornical organ) do not mediate the observed responses during acute increases in total body NaCl. We acknowledge that the potential impact of the AV3V on ANP release (Antunes-Rodrigues et al., 1992) during a 1M NaCl infusion was not examined in these studies. However, given that an AV3V lesion did not impact the observed physiological responses to a 1M NaCl infusion we speculate that AV3V mediated release of ANP does not play a major role in this setting. It is possible that paraventricular nucleus (PVN)-specific sodium sensitive pathways (Frithiof et al., 2009) including the intrinsic osmosensitive magnocellular neurons that are present in the PVN and supraoptic nucleus (Prager-Khoutorsky and Bourque, 2015) or peripheral sodium sensitive mechanisms [e.g., hepato-portal sodium response (Morita et al., 1997)] may contribute to the observed sympathoinhibitory responses to the 1M NaCl infusion. Additionally, we acknowledge that the neurohypophysial secretion of oxytocin and vasopressin plays a central role in fluid and electrolyte homeostasis (Mecawi Ade et al., 2015), and was not addressed in the current studies due to the confounding impact of collecting sufficient repeated blood volumes to assess circulating levels of oxytocin and vasopressin during a 1M NaCl infusion and may be investigated in future studies.

Given that our data demonstrate a central role of the renal sympathetic nerves in the natriuretic response to an acute 1M NaCl infusion we next investigated the potential signaling pathways through which this may be occurring. It is well established that the renal sympathetic nerves influence renal sodium excretion via two predominant mechanisms: norepinephrine-mediated stimulation of renal α_1_-adrenoceptors, evoking sodium reabsorption, or renal β1-adenoceptors, stimulating renin release (DiBona and Kopp, 1997). As such, we elected to investigate the impact of acute systemic α_1_- and β-adrenoceptor antagonism during an acute 1M NaCl load. A 1-h infusion of the α_1_-adrenoceptor antagonist terazosin, prior to administration of a 1M NaCl load, resulted in a reduction in arterial blood pressure (likely mediated by antagonism of vascular α_1_-adrenoceptors) and an increase in heart rate. Further, α_1_-adrenoceptor antagonism suppressed baseline diuresis and free water. Similar to our observations in in naïve and RDNX rats, a 1M NaCl did not impact cardiovascular parameters in rats receiving an α_1_-adrenoceptor antagonist infusion. Significantly, α_1_-adrenoceptor antagonism dramatically attenuated the natriuretic and diuretic responses to a 2-h 1M NaCl infusion – suppressing peak natriuresis to a similar magnitude as observed following acute RDNX. Also consistent with the impact of acute bilateral RDNX, α_1_-adrenoceptor antagonism resulted in an increase in estimated plasma volume without altering plasma sodium content during the 1M NaCl infusion. In contrast to our findings in RDNX rats, plasma volume remained elevated throughout the recovery period in rats receiving an α_1_-adrenoceptor antagonist infusion. This difference may reflect the continuous administration of an exogenous α_1_-adrenoceptor antagonist during the recovery period versus a temporary endogenous response evoked by the 2 h 1M NaCl infusion that was reversed during the recovery period in acutely RDNX animals. Despite the profoundly attenuated natriuretic response observed in animals receiving the α_1_-adrenoceptor antagonist infusion, we observed no increase in plasma sodium content. A possible explanation for this is the significantly greater decrease in free water clearance and increase in estimated plasma volume observed during sodium infusion compared with naïve rats, as these observations are consistent with enhanced renal water retention to maintain stable plasma sodium levels.

As noted, in our conscious rat model, acute α_1_-adrenoceptor antagonism evokes a reduction in baseline blood pressure prior to administration of a 1M NaCl infusion. We acknowledge it is possible that this alteration in blood pressure influenced the observed attenuated natriuretic response. This may have occurred by alterations in renal medullary blood flow, which was not assessed in these studies, and may occur despite no detectable changes in renal blood flow in response to the 1M NaCl load. To address the potential confounding impact of the observed significant drop in blood pressure during acute α_1_-adrenoceptor antagonism experimentally, we investigated the effects of α_1_-adrenoceptor antagonism via s.c. terazosin infusion at a dose we have previously published does not impact blood pressure (Frame et al., 2019b; Puleo et al., 2020). Matching our data prior data (Frame et al., 2019b; Puleo et al., 2020), s.c. infusion of terazosin had no impact on baseline blood pressure and fully and selectively antagonized the response to an α_1_ agonist. Consistent with our data in rats that underwent an acute terazosin infusion we observed was no change in cardiovascular parameters but a profound attention of the natriuretic response to a 1M NaCl load that was accompanied by increased blood and plasma volume. Notably renal denervation did alter the impact of α_1_-adrenoceptor antagonism on attenuating the natriuresis to a 1M NaCl load. Collectively, these data suggest a blood pressure-independent role for α_1_-adrenoceptors in the natriuretic response to an acute increase in total body NaCl.

To assess the potential role of β-adrenoceptors in the natriuretic pathways activated during acute increases in total body sodium, conscious rats were administered an infusion of the β-adrenoceptor antagonist propranolol. Reflecting the established effect of β1-adrenoceptors in the heart, β-adrenoceptor antagonism resulted in a reduction in baseline heart rate, but had no effect on blood pressure or renal excretory parameters. In rats receiving a β-adrenoceptor antagonist infusion a 1M NaCl-infusion which did not alter cardiovascular parameters, we observed preservation of the profound natriuretic and diuretic responses observed in naïve rats and estimated blood and plasma volumes remained constant during the experimental protocol. Our current findings of a lack of role of β-adrenoceptors in the acute natriuretic responses to non-pressor 1M NaCl infusion are supported by studies in conscious dogs and humans indicating that the natriuretic response to a NaCl load that does not alter blood pressure remains intact during β1-adrenoceptor antagonism (Bie et al., 2009; Molstrom et al., 2009). Together, these data strengthen our hypothesis that there is a pivotal role for renal sympathetic nerve activated α_1_-adrenoceptors in mediating the natriuresis during an acute challenge to total body NaCl homeostasis.

In light of the established mechanism whereby suppression of renal sympathetic outflow increases renal sodium excretion via reduced norepinephrine-α_1_-adrenoceptor-driven sodium reabsorption at the level of the kidney, our findings appear paradoxical. Studies to elucidate the mechanisms underlying the observed attenuation in natriuresis remain beyond the scope of the current manuscript. However, there are alternative extra-renal and renal actions of α_1_-adrenoceptors that could drive the observed clear attenuated natriuretic responses following α_1_-adrenoceptor antagonism. A potential extra-renal pathway through which α_1_-adrenoceptor antagonism, or a sympathoinhibitory reno-renal reflex, may reduce natriuresis is via a decrease in α_1_-adrenoceptor mediated release of atrial natriuretic peptide (Luchner and Schunkert, 2004). Despite the current and prior studies by our group illustrating that a 1M NaCl infusion reduces sympathetic outflow, there remains the possibility that NaCl, when first sensed by the body, trigger rapid, short term sympathoexcitation to the heart, resulting in α_1_-adrenoceptor-mediated release of atrial natriuretic peptide and natriuresis. An alternative hypothesis involves the renal nerves, as acute bilateral renal denervation attenuated natriuresis to approximately the same magnitude as that seen following acute pharmacological blockade of α_1_-adrenoceptors during a 1M NaCl infusion and RDNX in combination with pharmacological blockade of α_1_-adrenoceptors did not further impact the attenuation in natriuresis. These data support a role of the renal nerves in driving the natriuretic response to the infused 1M NaCl load. Additionally, it has been reported that renal pelvic α_1_-adrenoceptors present on the renal sensory afferent fibers respond to pharmacological and physiological stimuli that include norepinephrine and increased efferent renal sympathetic nerve activity (Kopp et al., 2007). To directly test the role of renal sensory afferent α_1_-adrenoceptors on the natriuretic responses to acute sodium, additional studies beyond the scope of this investigation in anesthetized rats in which α_1_-adrenoceptor antagonists are administered by a renal pelvic infusion are required.

## Conclusion

In conclusion, the present studies extend our knowledge of the renal sympathetic nerve mediated adrenoceptor-dependent signal transduction pathways that regulate natriuretic responses to acute increases in total body sodium in the absence of activation of the pressure-natriuresis mechanism. A key finding is that the α_1_-adrenoceptor is the major receptor system involved in facilitating natriuresis to maintain fluid and electrolyte homeostasis in response to acute sodium challenges in which blood pressure remains stable. In contrast to the established roles of the renal sympathetic nerves on renal sodium handling via renal α_1_-adrenoceptor-evoked sodium reabsorption and renal β1-adenoceptors-mediated renin release, our studies indicate that α_1_-adrenoceptor activation is required for the acute renal sympathetic nerve-dependent natriuretic response to increases in total body NaCl. Given the proposed roles of α- and β-adrenoceptors on the chronic regulation of the sodium chloride cotransporter and long term sodium homeostasis (Mu et al., 2011; Terker et al., 2014; Frame et al., 2019b; Puleo et al., 2020) our findings suggest differential roles of the actions of the renal sympathetic nerves via α_1_- and β-adrenoceptors during acute versus chronic challenges to sodium homeostasis, potentially via a sympathoinhibitory reno-renal reflex in the acute setting. These findings are physiologically relevant as increased understanding of the roles of the renal sympathetic nerve mediated mechanisms impacting the acute natriuretic responses to elevations in total body sodium has implications for the mechanisms underlying multiple pathophysiological states featuring sodium retention, e.g., heart failure, salt-sensitive hypertension.

## Data Availability Statement

The original contributions presented in the study are included in the article/supplementary material, further inquiries can be directed to the corresponding author.

## Ethics Statement

The animal study was reviewed and approved by Boston University IACUC Committee.

## Author Contributions

AF, JK, and RW performed experiments. RW prepared figures. AF, KN, KK, and RW drafted manuscript. All authors approved final version of manuscript.

## Conflict of Interest

The authors declare that the research was conducted in the absence of any commercial or financial relationships that could be construed as a potential conflict of interest.

## Publisher’s Note

All claims expressed in this article are solely those of the authors and do not necessarily represent those of their affiliated organizations, or those of the publisher, the editors and the reviewers. Any product that may be evaluated in this article, or claim that may be made by its manufacturer, is not guaranteed or endorsed by the publisher.
